# Simple Control

**DOI:** 10.5334/joc.107

**Published:** 2020-09-10

**Authors:** Nachshon Meiran

**Affiliations:** 1 Department of Psychology and Zlotowski Center for Neuroscience, Ben-Gurion University of the Negev, Beer-Sheva, IL

**Keywords:** Attention, Executive functions, Cognitive Control

Schmidt, Liefooghe and De Houwer (2020, henceforth, SLD) present an impressive theoretical work, which suggests a novel perspective on task-switching behavior and also shows its unique contribution relative to other models. In a nutshell, SLD show that switching-cost, believed to be an empirical marker of cognitive control, can be explained in terms of simple episodic binding.

Models serve for *model-based estimation* of latent variables (e.g., Signal Detection Theory, McMillan, 2004, enabling the estimation of sensitivity and bias) and as *proofs of concept* (e.g., SLD’s model, showing that episodic binding can explain switching cost), thereby clarifying the necessary assumptions in a scientific explanation. I address these two aspects in turn.

Estimation of latent variables is widespread in cognitive psychology, with subtraction (also employed to compute switch cost) being probably the most widely used model. As usual, the estimate (e.g., switch cost) is valid as long as the underlying model is approximately valid. To appreciate this point, consider a *hypothetical* model assuming that task-switch trials entail reconfiguration, a switch-unique *proactive* processing stage that precedes response selection. Under this model, *switch-cost = RT_switch_* – *RT_non-switch_* provides an estimate for the duration of the (latent) reconfiguration processing stage. We however already know this model to be inaccurate because (a) residual switch cost, i.e. switch cost observed after ample advance task-preparation, is often observed, suggesting the involvement of additional processes beyond reconfiguration; and (b), the slope describing the reduction in switch cost as a function of task preparation time is far shallower than –1 (see Pashler, 1994). This finding suggests that reconfiguration, at minimum, is very slow and inefficient when performed ahead of the imperative stimulus, a fact that seems to argue against the proactivity hypothesis in general. SLD provide an alternative account of switch costs but seem to also suggest that switch costs do not represent cognitive control but represent what they describe as simpler memory mechanisms. I doubt this conclusion and will suggest one challenging fact: the increased switch cost observed in attention deficits. This finding that has been replicated several times, including a related finding of “normalization” of switch costs under methylphenidate treatment (Kramer, Cepeda, & Cepeda, 2001; Luna-Rodriguez, Wendt, Kerner auch Koerner, Gawrilow, & Jacobsen, 2018; Rauch, Gold, & Schmitt, 2012). Given how attention deficits are defined by the DSM (American Psychiatric Association, 2013), it is difficult to attribute the increased switch cost and its “normalization” to features that are completely unrelated to cognitive control. A possible solution is that switch cost is related to *reactive* control rather than proactive control (Braver, Reynolds, & Donaldson, 2003), implying that attention deficits reflect a difficulty in reactive control (Grane et al., 2016). SLD’s model suggests another hypothesis regarding an indirect link. According to it, people who are characterized by attention deficits compensate for it by reliance on episodic memory, which results in increased switching costs.

I now turn to the use of models as a proof of concept. An important advantage of this use is the requirement to specify necessary assumptions and clarify their explanatory contribution. As acknowledged by SLD, their model had to incorporate control-related features including serial processing, abstract task representations, instructions, and the recall of instructions after errors (SLD, Appendix B). In Duncan’s (2010) “general demand network”, *serial processing* requires setting the processing sequence and monitoring its progress. In Logan and Gordon’s (2001) ECTVA model, the function of serial processing to reduce crosstalk and errors, and accordingly, increased control demands are accompanied by a shift to serial processing (Luria & Meiran, 2005). Arguably, as a result of insufficiently *abstract task representations*, toddlers fail task switching, and “get stuck” on a single task (Zelazo, 2004), and when not focusing on abstract task representations, adult participants, like pigeons (Castro & Wasserman, 2016) and monkeys (Avdagic, Jensen, Altschul, & Terrace, 2014; Stoet & Snyder, 2003) perform the task switching task as if it were a single task and do not show switch costs (Dreisbach, Goschke, & Haider, 2006, 2007). Moreover, abstract task representations, like serial processing, reduce crosstalk and interference (Dreisbach, 2012). Interestingly, the evolution of the primate cortex resulted in us having association cortices that are remote from primary sensory systems, enabling representational abstractness and behavioral flexibility (Kaas & Herculano-Houzel, 2017). Along a similar line, the posterior (close to primary sensory)-to-anterior (remote from primary sensory) axis in the human prefrontal cortex also reflects a representational shift from concrete to abstract (Koechlin & Summerfield, 2007; O’Reilly, 2010). These observation suggest that *instructions* in a very specific (abstract) format are an essential requirement for task switching to happen and switch cost to emerge. The *recall of instructions after errors* may in fact underlie the control-related, and especially pronounced post-error slowing observed in the task-switching task (Regev & Meiran, 2014). Although SLD’s model suggests that task-switching involves many aspects of control, it does not suggest an involvement of the key control element of working memory. This conclusion seems to be in line with the fact that following *novel* instructions is impaired by working memory load (Pereg & Meiran, 2019). However, working memory seems to be relatively minimally involved in the task-switching task, in which the same instructions are executed multiple times (e.g., Kessler & Meiran, 2009; Rubin & Meiran, 2005; van’t Wout, Lavric, & Monsell, 2013).

My conclusions are that contrary to a widely held belief, “automatic” and “episodic” are not alternatives to cognitive control. For example, automatic effects may reflect side effects of control (Meiran, Liefooghe, & De Houwer, 2017). Thus, as suggested by SLD’s model, it is the joint operation of “simple” processes of episodic memory and the aforementioned control features that enable cognitive control operations to occur.
